# Amines as key building blocks in Pd-assisted multicomponent processes

**DOI:** 10.3762/bjoc.7.163

**Published:** 2011-10-10

**Authors:** Didier Bouyssi, Nuno Monteiro, Geneviève Balme

**Affiliations:** 1 ICBMS, Institut de Chimie et Biochimie Moléculaire et Supramoléculaire, CNRS UMR 5246, Université Lyon 1, ESCPE Lyon. 43, Bd du 11 Novembre 1918, 69622, Villeurbanne, France

**Keywords:** amines, multicomponent reactions, palladium

## Abstract

In the last few years, palladium-mediated three-component synthesis has emerged as an important synthetic methodology to gain access to nitrogen-containing structures. The latest developments in this area are discussed in this review.

## Introduction

Nitrogen-containing structures are present in numerous bioactive natural and synthetic products. The development of new methodologies to prepare these useful frameworks has attracted great attention from organic chemists. Among these developments, multicomponent strategies offer significant advantages over stepwise procedures since several bonds are formed in a one-pot operation, minimizing the formation of waste and competitive reactions [1]. In line with this, remarkable new strategies have been developed based on palladium-mediated coupling process. The purpose of this review is to discuss recent achievements in the design of palladium-catalyzed multicomponent preparation of nitrogen-containing structures and this article is divided into sections relating to the introduction of the amine functionality.

## Review

### Imines as electrophilic partners

The imine function plays an important role in the development of multicomponent approaches to polyfunctionalized nitrogen acyclic or cyclic compounds due to the ease of their in situ preparation. Many strategies have been developed based on this concept, the imines being either directly used as starting building blocks or generated in situ as part of the multicomponent process.

**Scheme 1:**
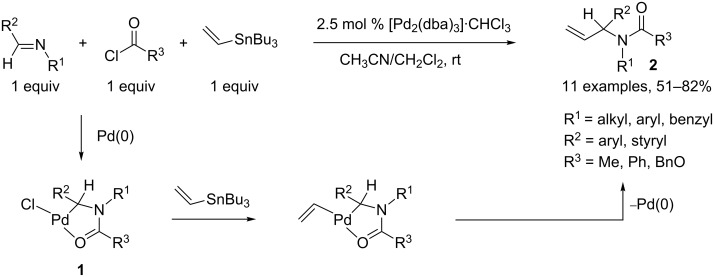
Synthesis of substituted amides.

Arndtsen and coworkers elaborated a three-component process allowing the synthesis of α-substituted amides. This methodology relied on the oxidative addition of an *N*-acyliminium species, generated in situ from an imine and an acid chloride, to a Pd(0) complex, furnishing a stable chelated palladium adduct **1**, which was isolated and fully characterized. When vinyltributyltin was added as a third component in the reaction medium, a transmetallation step occurred, followed by a reductive elimination step, furnishing amides **2** in good to excellent yields [2]. This reaction tolerated various functional groups on the imine moiety, such as ether, thioether and ester groups, although enolizable alkylimines were not suitable under these conditions (Scheme 1).

**Scheme 2:**
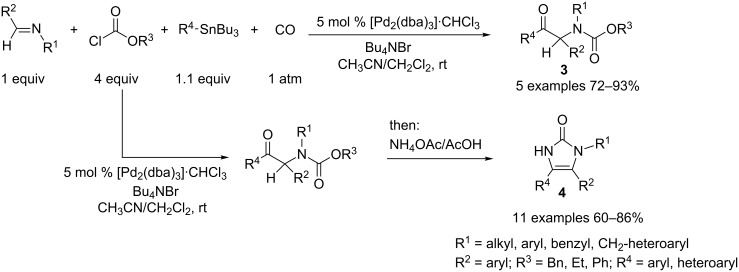
Synthesis of ketocarbamates and imidazolones.

Replacement of the acid chloride with a chloroformate under 1 atmosphere of carbon monoxide as a fourth component led to ketocarbamates **3** in a single operation through a carbonylative coupling [3]. Various chloroformates and imines can participate in this reaction, stannanes being limited to aryl, benzyl or ethyl ones. When vinylstannane was used, the transmetallation step was more rapid than the CO insertion, giving instead substituted carbamates. After removal of the solvents in vacuo, the addition of acetic acid and 15 equivalents of ammonium acetate to the crude mixture resulted in a postcyclization leading to imidazolones **4**, with spontaneous elimination of the initial chloroformate substituent (Scheme 2).

**Scheme 3:**
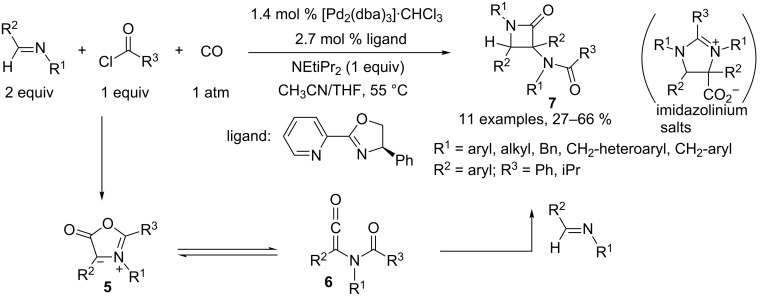
Access to β-lactams.

Arndtsen also demonstrated that 3-amido-substituted β-lactams **7** can be obtained, based on a similar strategy, through the assembly of four components, namely, imines, acid chloride and carbon monoxide. The process is thought to begin with formation of a münchnone **5**, resulting from oxidative addition of an acyliminium species to Pd(0), followed by CO insertion and β-hydride elimination. This münchnone is in equilibrium with its ketene isomeric form **6**, and a formal [2 + 2] cycloaddition with a second equivalent of imine generates the lactam (Scheme 3). The authors pointed out that the trapping of HCl by a sterically hindered base (NEtiPr_2_) is the key point in this methodology to enable access to this heterocycle and to avoid formation of imidazolinium salts [4].

**Scheme 4:**
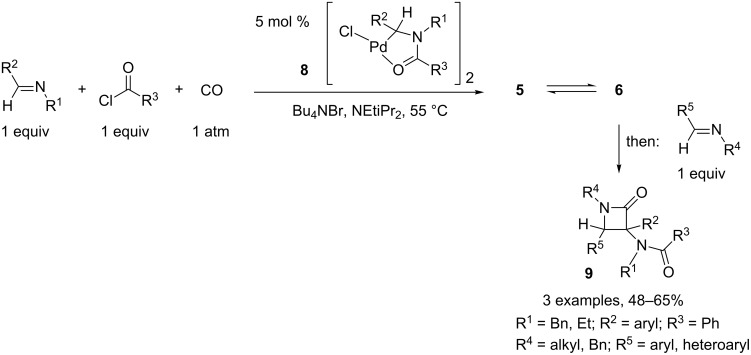
Access to β-lactams with increased structural diversity.

To increase the structural diversity of the final lactam, a modified process was developed that allows introduction of two distinct imines in this reaction. This coupling process was catalyzed by palladacycle **8** and led to the mesoionic compound **5** under these conditions. Subsequent addition of a second, different imine produced β-lactams **9** in good yields, after heating at 55 °C for 24 h (Scheme 4) [4].

**Scheme 5:**
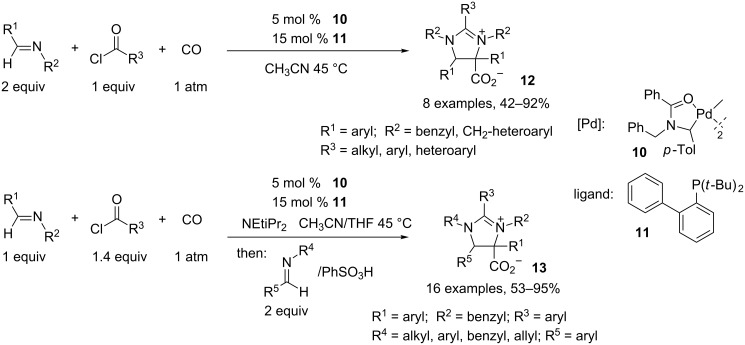
Synthesis of imidazolinium salts.

As mentioned above, imidazolinium salts **12** can be obtained by a dipolar cycloaddition of münchnone intermediates with imines. Arndtsen and coworkers developed a new highly active palladium catalyst to improve previous results in this area. Moreover, this strategy allows the selective incorporation of two different imines leading to polysubstituted imidazoliniums **13**. After a large screening of palladium precatalysts and ligands, the palladacycle **10** in combination with the di-*tert*-butyl-2-biphenylphosphine (**11**) furnished the best results in terms of reaction time and yield. A large variety of imines and acid chlorides can be used in this reaction, with only enolizable imines and those bearing bulky nitrogen substituents being incompatible. In order to have four independent tunable substrates, the authors added a base (NEtiPr_2_) to the reaction medium that favors formation of the münchnone intermediate. The second imine was added after 16 h of heating at 45 °C, together with PhSO_3_H, which catalyzed the dipolar cycloaddition and avoided formation of a β-lactam as shown before (Scheme 5) [5].

**Scheme 6:**
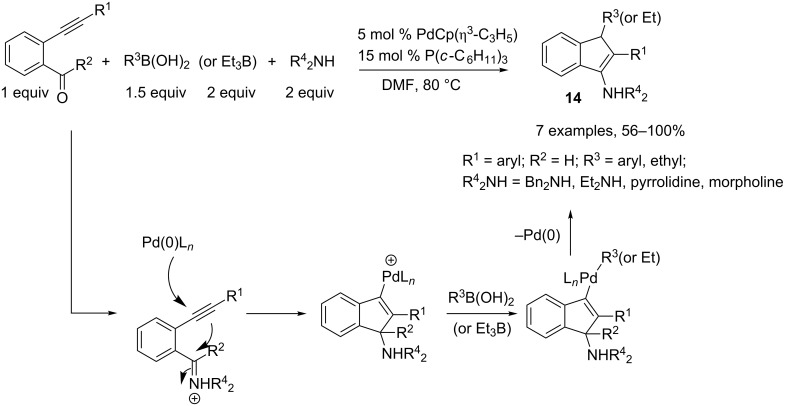
Access to the indenamine core.

The palladium-catalyzed trans-addition-alkylative cyclization (anti-Wacker cyclization) of *o*-ethynylbenzaldehyde with organoboron reagents in the presence of secondary amines was accomplished by Tsukamoto and coworkers [6]. This novel strategy, dedicated to the synthesis of indenamines **14**, involves addition of an electron-rich palladium/phosphine complex to a triple bond, followed by nucleophilic addition to an iminium ion generated in situ by addition of a secondary amine to an aldehyde. Transmetallation of the resulting species with a boronic acid or triethylborane, followed by a reductive elimination, afforded the indenamine core in good to excellent yields. However, this Pd-catalyzed cyclization was only effective for aldehydes since ketones did not participate in the process (Scheme 6).

**Scheme 7:**
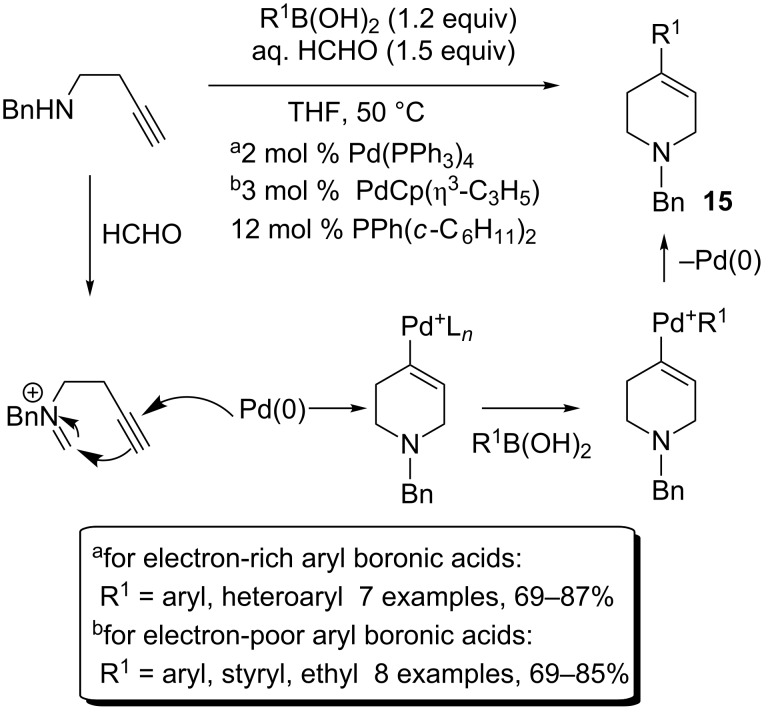
Synthesis of substituted tetrahydropyridines.

Tsukamato extended this methodology further to the cyclization of alkynyl- and allenyliminiums in order to access 1,4-disubstituted-1,2,3,6-tetrahydropyridines **15** or **16** following the same strategy [7]. For alkynyliminiums, two different catalytic systems were developed according to the nature of the aryl- or heteroarylboronic acids used. For neutral or electron-rich acids, Pd(PPh_3_)_4_ as catalyst gave excellent results, whereas it was necessary to use PdCp(η^3^-C_3_H_5_) in the presence of PPh(*c*-C_6_H_11_)_2_ as a ligand for those bearing electron-withdrawing groups (Scheme 7).

**Scheme 8:**
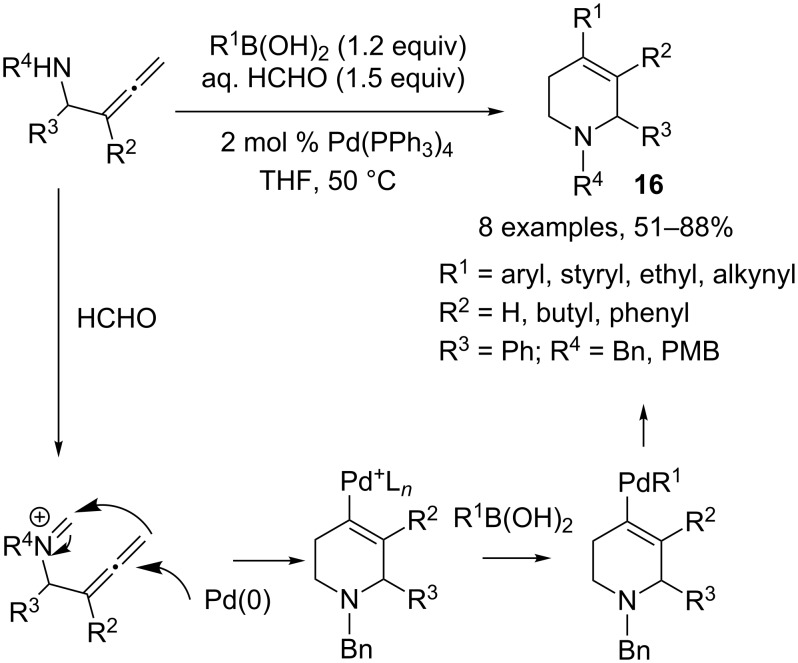
Synthesis of more substituted tetrahydropyridines.

The proposed mechanism involves addition of Pd(0) onto the triple bond, followed by nucleophilic attack on the iminium generated in situ. The resulting vinylpalladium species reacts with the boron or alkynyl compound as previously shown. Allenylamines are also compatible with this anti-Wacker process, leading to more substituted tetrahydropyridines **16** in good to excellent yields (Scheme 8).

**Scheme 9:**
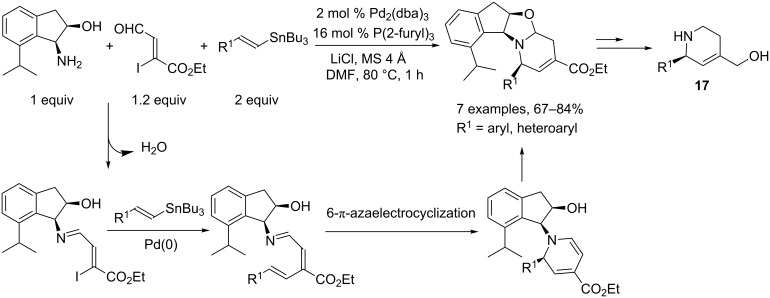
Synthesis of chiral tetrahydropyridines.

Another strategy allowing access to pyridine derivatives was developed by Katsumura and coworkers. They showed that chiral 2,4-disubstituted 1,2,5,6-tetrahydropyridines **17** can be obtained through a one pot imine synthesis, Stille coupling, 6π-azaelectrocyclization and aminoacetal formation. The chiral auxilliary can be removed by further treatment with DIBAL-H and Pb(OAc)_4_ (Scheme 9) [8].

**Scheme 10:**
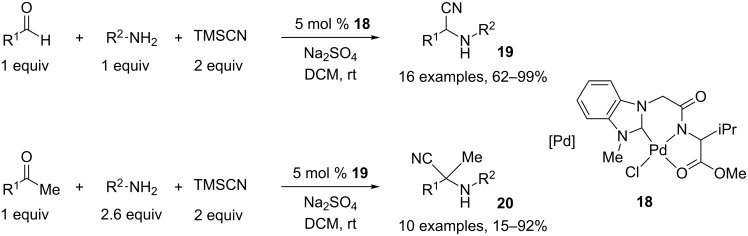
Preparation of α-aminonitrile by a catalyzed Strecker reaction.

The Strecker reaction, employing aldehydes or ketones and a cyanide source, is a very useful route for the preparation of α-aminonitriles **19** or **20**. A general and efficient three-component method was reported by Jung and coworkers who used a new catalytic system based on a NHC–amidate palladium(II) complex **18**. This complex acts as a Lewis acid to favor addition of cyanide to the imine generated in situ. This methodology employs smooth conditions and works with aldehydes as well as ketones, giving good to excellent yields (Scheme 10) [9].

**Scheme 11:**
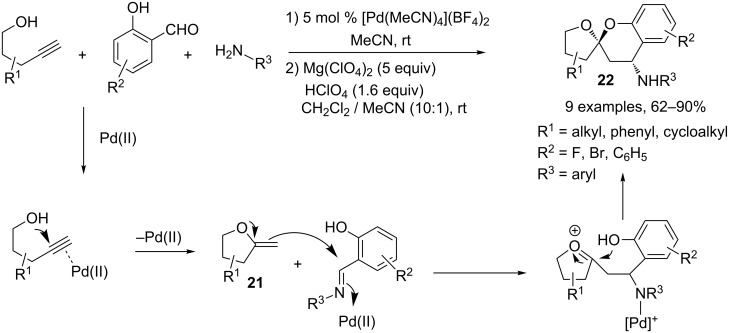
Synthesis of spiroacetals.

Barluenga and coworkers reported a synthesis of spiroacetals **22**, through a Pd(II)-catalyzed three-component cascade reaction, starting from an alkynol, an aldehyde and a primary amine. The authors suggested that the first step of the reaction was the attack of the hydroxyl group onto the triple bond activated by a Pd(II) cationic complex, followed by a protodemetalation, which afforded the methylidenefuran **21**. This reacts with the imine activated by the Pd(II) species through a Mannich-type process. Finally, addition of the phenol to the oxonium can lead to spiroacetal **22**. One major drawback of this MCR is the formation of an equimolar amount of two diastereomers, which can be circumvented by further treatment of the crude mixture with 5 equivalents of MgClO_4_ and 1.6 equivalents of HClO_4_ in CH_2_Cl_2_/MeCN at room temperature. Under these acidic conditions, one diastereomer was cleanly and completely transformed into the other one (Scheme 11) [10].

**Scheme 12:**
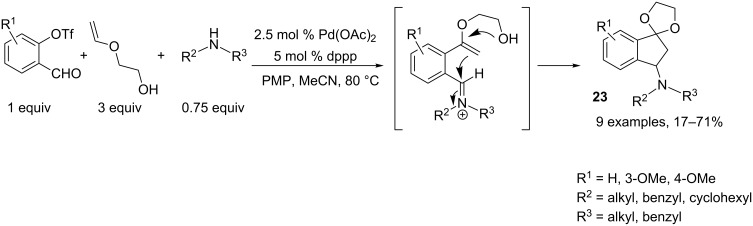
Synthesis of masked 3-aminoindan-1-ones.

Hallberg and coworkers developed a one-pot strategy towards the synthesis of masked 3-aminoindan-1-ones **23**. This process was initiated by Heck addition of an aryl triflate to a vinyl ether, leading to an α-arylation product, followed by iminium formation in the presence of a secondary amine and subsequent tandem cyclization. The authors showed the importance of the ratio of the diverse reactants, notably that the amount of amine should remain low to avoid formation of aminal derivatives that would block the ring closure (Scheme 12) [11].

**Scheme 13:**
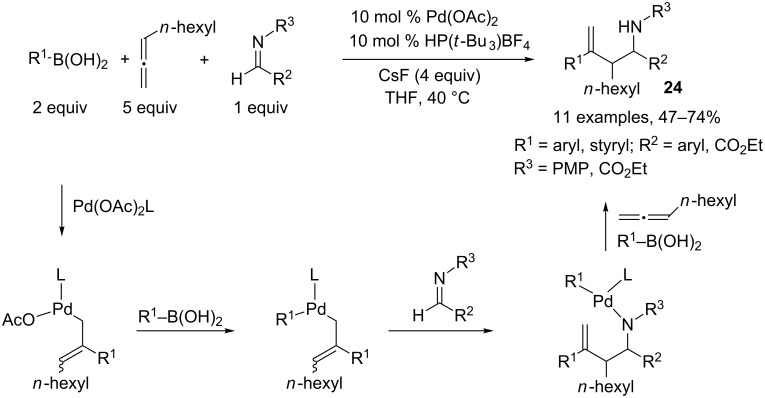
Synthesis of homoallylic amines and α-aminoesters.

Homoallylic amines and α-aminoesters **24** were prepared by Malinakova and coworkers, by a palladium(II)-catalyzed coupling of boronic acids, 1,2-nonadiene, and aliphatic, aromatic or heteroaromatic imines [12]. The authors postulated a transmetalation step between the Pd(II) complex and a boronic acid activated by CsF, followed by insertion of the resulting σ-arylpalladium(II) into the allenic moiety leading to a π-allyl intermediate. This can undergo a nucleophilic allyl transfer to the imine, generating an amino-Pd(II) complex, which can subsequently add to another allenic unit. After a new transmetalation step with the boronic acid, the active catalytic species can be released and entered into a new catalytic cycle (Scheme 13).

### Imine as a nucleophilic partner

**Scheme 14:**
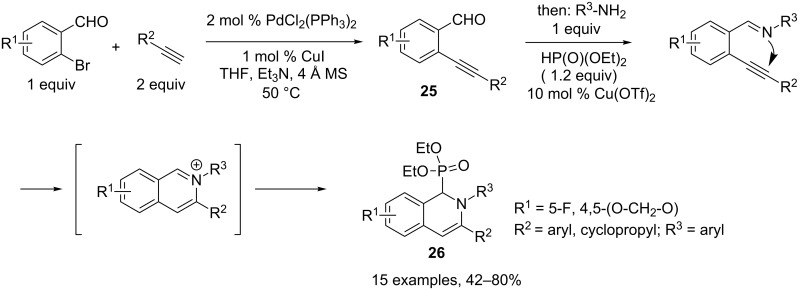
Preparation of 1,2-dihydroisoquinolin-1-ylphosphonates.

A tandem four-component reaction allowing access to 1,2-dihydroisoquinolin-1-ylphosphonates **26** was reported by Wu and coworkers. Initial Sonogashira coupling was effected between a 2-bromobenzaldehyde and an alkyne in the presence of catalytic amounts of PdCl_2_(PPh_3_)_2_ and CuI. After complete conversion of the aldehyde into the coupling product **25** (TLC control), a primary amine and diethylphosphite were added to the reaction medium with concomitant addition of 10 mol % of Cu(OTf)_2_ necessary to complete the cyclization step. The proposed mechanism involves formation of an imine intermediate, which attacks the triple bond activated by the copper(II) complex. The resulting iminium was finally trapped by addition of diethylphosphite. Moderate to good yields were obtained depending on the nature of the various components (Scheme 14) [13].

### Amines as hetero-Michael donors

**Scheme 15:**
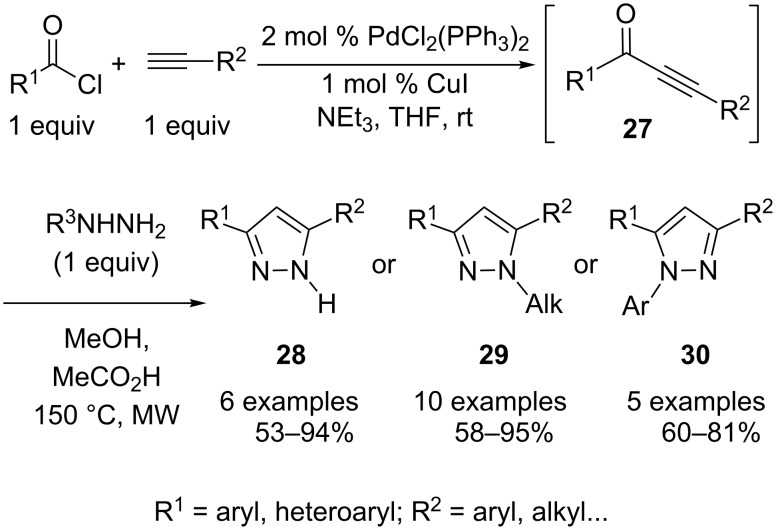
Pyrazole elaboration by cycloaddition of hydrazines with alkynones generated in situ.

Many multicomponent approaches to nitrogen heterocycles have been developed based on the reaction of nitrogen-centered nucleophiles with α,β-unsaturated ketones generated in situ by Pd-catalyzed Sonogashira cross-coupling reactions. For instance, by building on their expertise in this area [14] Müller and coworkers recently developed a very effective and modular three-component strategy to assemble a series of 3,5-bis(hetero)aromatic pyrazoles in a consecutive fashion from terminal alkynes, acid chlorides, and hydrazine derivatives. Classical approaches to these valuable compounds are notably based on the cyclocondensation of hydrazine derivatives with 1,3-disubstituted three-carbon units, including α,β-unsaturated ketones, and particularly alkynones. In situ generation of the latter is an interesting means of overcoming the poor commercial availability of these compounds and also offers the flexibility needed for library production (Scheme 15). Thus various (hetero)aryl acid chlorides and terminal alkynes were heated in THF in the presence of Et_3_N and catalytic amounts of PdCl_2_(PPh_3_)_2_ and CuI. The resulting ynones **27** were then treated in situ with diversely substituted hydrazine derivatives to produce, upon microwave heating, a series of pyrazoles **28**–**30** (Scheme 15). As previously established for this type of cycloaddition, one of the two possible regioisomers was obtained preferentially depending on the hydrazine derivatives used, *N*-alkyl- and *N*-arylhydrazines giving opposite regioselectivities [15].

**Scheme 16:**
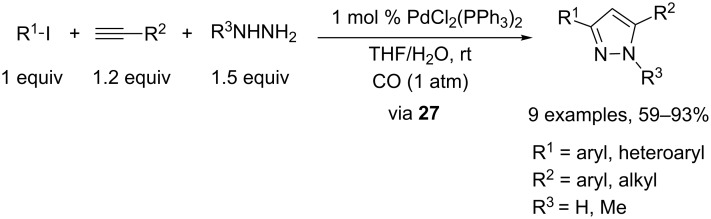
An alternative approach to pyrazoles involving hydrazine cycloaddition.

The carbonylative coupling of terminal alkynes with aryl (and heteroaryl) halides was proposed by Mori and coworkers as a different approach to α,β-alkynyl ketone derivatives as pyrazole precursors. They established a four-component domino process combining various organic halides, terminal alkynes, hydrazines, and carbon monoxide at room temperature. In this case, all components are mixed at the very beginning of the process, in aqueous THF, under ambient pressure of CO and in the presence of 1 mol % PdCl_2_(PPh_3_)_2_ as the sole catalyst. However, one drawback of this approach is that it is, so far, limited to simple hydrazine and *N*-methylhydrazine (Scheme 16). From a mechanistic point of view, it is interesting to note that the intermediacy of α,β-alkynyl ketones in the four-component process could not be confirmed (TLC). In addition, their reaction with hydrazines was shown to be ineffective under the present solvent system in the presence or absence of palladium catalyst. This may suggest that if α,β-alkynyl ketones are formed, they immediately react with hydrazine to form pyrazole by a specific rate acceleration in the one-pot process [16].

**Scheme 17:**
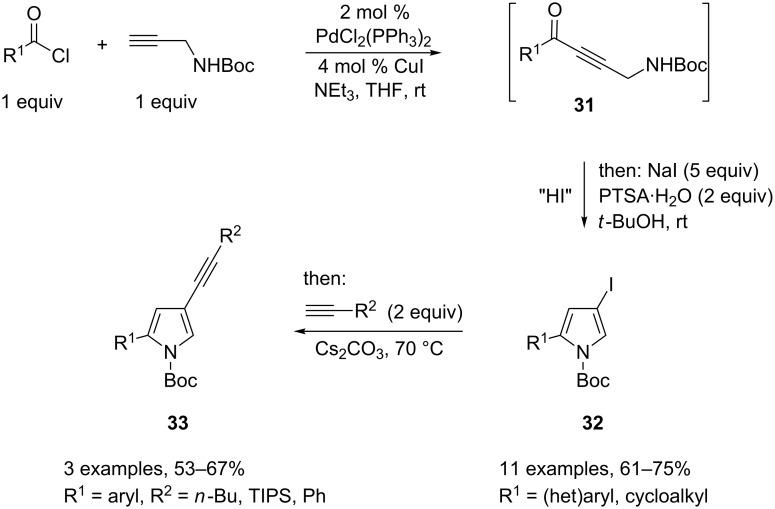
Synthesis of pyrroles by cyclization of propargyl amines.

The Sonogashira cross-coupling of acid chlorides with terminal alkynes has also been demonstrated as a valuable tool to generate, in situ, ynones bearing a pendant amine group **31**, which will undergo addition–intramolecular cyclocondensation processes leading to the formation of pyrrole derivatives. For instance, a series of (hetero)aryl-, alkynyl-, and cycloalkyl acid chlorides were cross-coupled with *N*-Boc-protected propargylamine at room temperature, and the resulting ynones were then treated in situ with sodium iodide and PTSA to yield 2-substituted *N*-Boc-4-iodopyrroles **32** in good overall yields. Interestingly, this product may be further transformed in situ into the corresponding *N*-Boc-4-alkynylpyrroles **33** by a further Sonogashira coupling that makes use of the still-operative palladium complex. To do so, a terminal alkyne and caesium carbonate were added to the reaction mixture containing the newly formed 4-iodopyrrole, and the reaction temperature was increased to 70 °C (Scheme 17) [17].

**Scheme 18:**
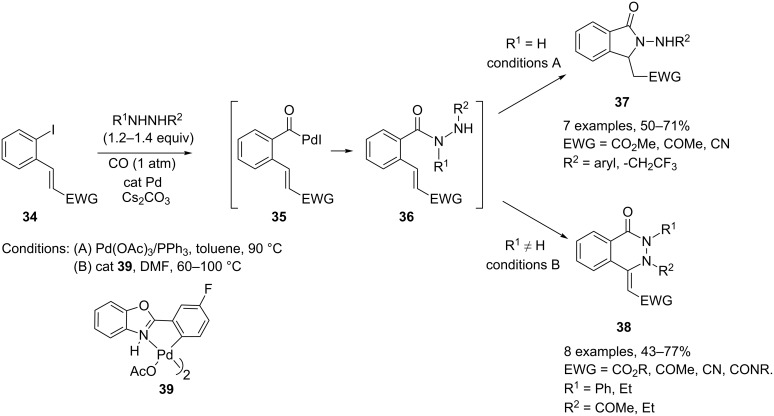
Isoindolone and phthalazone synthesis by cyclization of acylhydrazides.

Grigg and coworkers reported a three-component cascade process for the synthesis of isoindolones and phthalazones starting from *ortho***-**halogenated cinnamates **34** and related compounds in the presence of hydrazine derivatives and carbon monoxide. The process is thought to begin with carbonylation of the starting aryl iodide to give an acylpalladium species **35**, which is intercepted by the hydrazine nucleophile to give an acylhydrazide intermediate **36**. The latter undergoes intramolecular Michael addition to give either *N*-aminoisoindolones **37** or mono-*N*- and di-*N,N*′-phthalazones **38**, depending essentially on whether a monosubstituted or 1,2-disubstituted hydrazine derivative is used. A proper choice of catalyst and reaction conditions is also needed to improve the efficiency of each reaction (Scheme 18) [18].

**Scheme 19:**
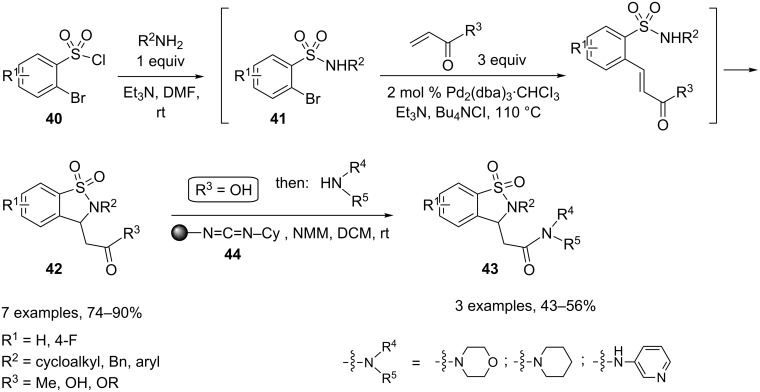
Sultam synthesis by cyclization of sulfonamides.

Consecutive one-pot transformations initiated by Heck reaction and terminated by intramolecular aza–Michael addition were developed by Hanson and coworkers to access a series of benzo-fused sultams. A range of α-bromobenzenesulfonyl chlorides **40** were first coupled with various amines in DMF at room temperature in the presence of Et_3_N to generate intermediate sulfonamides **41**. Subsequent in situ addition of a Michael acceptor in large excess together with Et_3_N, Bu_4_NCl, and catalytic Pd_2_(dba)_3_·CHCl_3_ led to the production of the desired sultams **42** upon heating at 110 °C. A series of sultam derivatives of bioactive, related isoindol-1-one amides **43** were also prepared by entering acrylic acid into the Heck–aza–Michael process and coupling a second amine derivative (after removal of excess acrylic acid) with the aid of an oligomeric alkyl carbodiimide **44** (Scheme 19) [19].

**Scheme 20:**
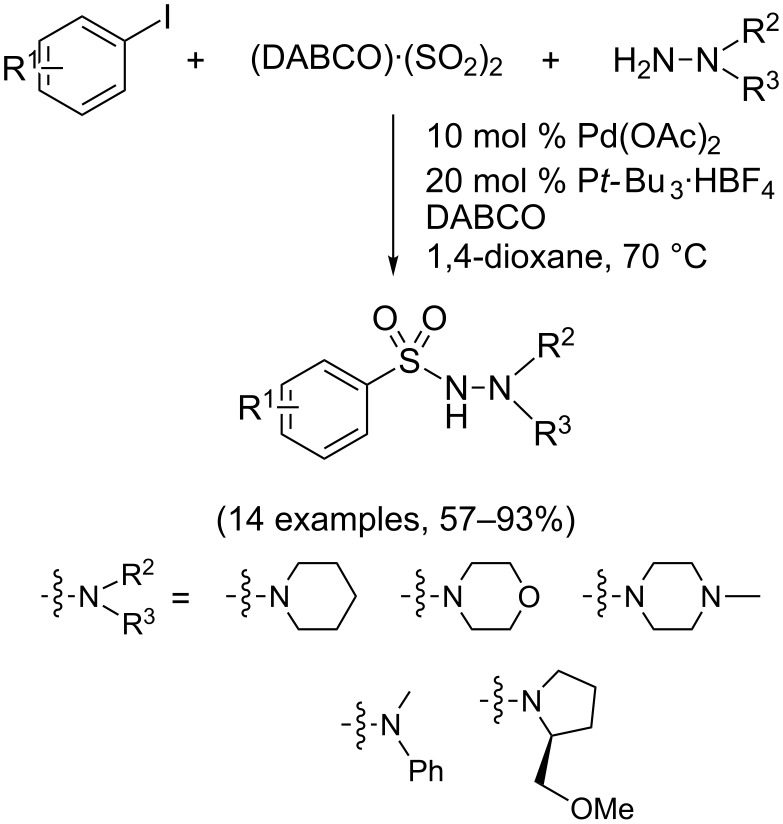
Synthesis of sulfonamides by aminosulfonylation of aryl iodides.

Interestingly, Willis and coworkers have shown that aryl *N*-aminosulfonamides may be accessed by three-component coupling of aryl iodides, hydrazines, and DABCO·(SO_2_)_2_ as a convenient source of sulfur dioxide. However, this Pd-catalyzed aminosulfonylation process proved inefficient with primary amines (Scheme 20) [20].

**Scheme 21:**
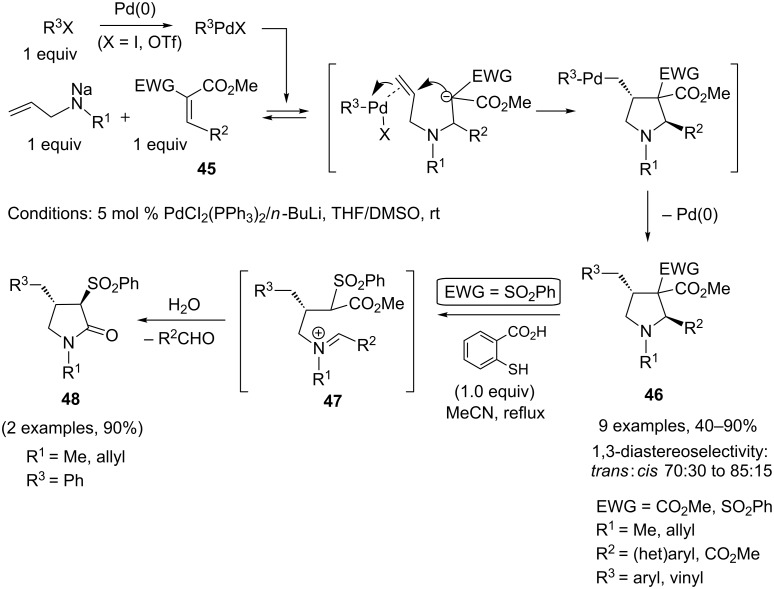
Pyrrolidine synthesis by carbopalladation of allylamines.

Multicomponent synthesis of nitrogen-containing heterocycles may also be initiated by an aza-Michael addition and terminated by a palladium-catalyzed ring-closure process [21]. For instance, Balme and coworkers reported a Pd-catalyzed three-component assembly of highly functionalized 4-benzyl- and allyl-pyrrolidines **46** based on a combination of allylamines (in situ transformed to their sodium salts by treatment with NaH), *gem*-diactivated alkenes **45** as Michael acceptors, and unsaturated halides (or triflate). Equal amounts of each of the three partners were reacted at room temperature in the presence of a catalytic quantity of a palladium(0) catalyst generated in situ by reduction of PdCl_2_(PPh_3_)_2_ with *n*-butyllithium. The key step in this one-pot transformation is the Pd-mediated cyclofunctionalization of the allyl moiety by carbopalladation/reductive elimination [22]. It is interesting to note that 3-sulfonylpyrolidin-2-ones (γ-lactams) **48** may also be accessed in high yield as single *trans*-diastereomers upon simple treatment of *N*-allyl- or *N*-methylpyrrolidines with 2-mercaptobenzoic acid in boiling MeCN. Acid-promoted formation of a ring-opened iminium salt intermediate **47**, followed by hydrolysis and subsequent intramolecular attack of the released secondary amine onto the ester group, would account for the formation of the γ-lactams [23]. This unexpected transformation was observed during attempted Pd-catalyzed deallylation of *N*-allyl-3-sulfonylpyrrolidines in the presence of 2-mercaptobenzoic acid according to the procedure developed by Genêt and coworkers [24] (Scheme 21).

### Amines as coupling partners through hydroamination of alkyne derivatives

**Scheme 22:**
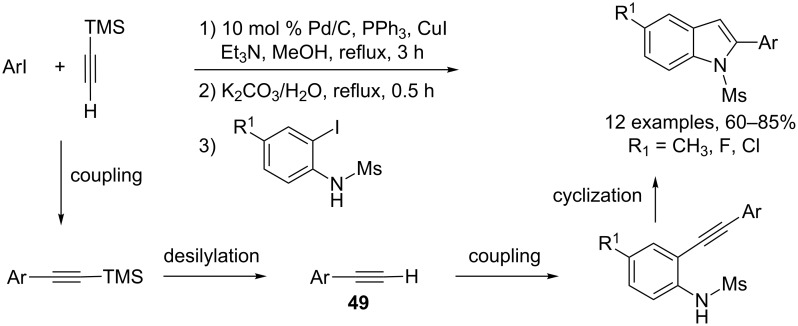
Synthesis of indoles through a sequential C–C coupling/desilylation–coupling/cyclization reaction.

Many synthetic methods for the preparation of indole derivatives have been reported because they occur in numerous natural products and bioactive compounds. Among these different strategies, those involving a palladium-catalyzed coupling reaction have received much attention [25] and one of the most commonly used procedures involves a one-pot two-step reaction with, first, a Sonogashira coupling of *o*-haloanilines with terminal alkynes, followed by a cyclization reaction of the resulting 2-alkynyaniline derivatives [26–27]. A strategy for the preparation of indoles through a three-component reaction consisted of generating the terminal alkyne precursor **49** in situ through a Pd/Cu mediated coupling reaction between (trimethylsilyl)acetylene (TMSA) with an aryl iodide, followed by a desilylation reaction. The subsequent addition of the third partner, an *o*-iodoanilide derivative, allowed a Pd/Cu tandem C–C/C–N-bond-forming reaction. The main advantage of this multicomponent reaction is to suppress the isolation of the pure form of the arylalkyne derivatives, which often represents a problem due to their ability to dimerize. This one-pot four-step reaction proceeded well with a series of electron-rich and electron-poor aryl iodide derivatives, and the best results were obtained when Pd/C–PPh_3_ was used as the catalyst system (Scheme 22).

**Scheme 23:**
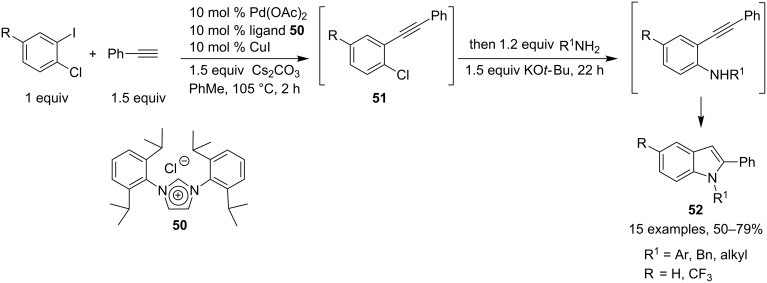
Synthesis of indoles by a site selective Pd/C catalyzed cross-coupling approach.

Another attractive palladium-mediated multicomponent approach towards the synthesis of indole derivatives involving the cyclization of a 2-alkynylaniline intermediate is based on a sequential, site-selective Pd-catalyzed cross-coupling approach starting from 1-chloro-2-iodobenzenes, phenylacetylene and a variety of primary amines [28–29]. The sequential three-component reaction was performed with the aid of an N-heterocyclic carbene-palladium complex generated in situ, derived from imidazolium salt **50** and Pd(OAc)_2_, and with CuI as the catalyst system. A first Sonogashira coupling reaction occurred, in the presence of Cs_2_CO_3_ as base, leading to ortho-alkynylchloroarene intermediates **51**. A subsequent amination was possible due to the high catalytic activity of this palladiumcarbene complex in the coupling of aryl chlorides. This was followed by an intramolecular alkyne–hydroamination (addition of an N–H bond across a carbon–carbon multiple bond) leading to the corresponding indole derivatives **52**. The amination/alkyne–hydroamination sequence requires the addition of 1.5 equiv of *t*-BuOK to reach completion. A variety of amines were involved in this one-pot sequential three-component reaction allowing the introduction of different protecting groups of the indole moiety. This site-selective, Pd/Cu-catalyzed cross-coupling approach was also performed on 1-chloro-2-iodo-4-(trifluoromethyl)benzene as *o*-dihaloarene partner and the corresponding polysubstituted indoles were isolated in good yields as single regioisomers (Scheme 23).

**Scheme 24:**
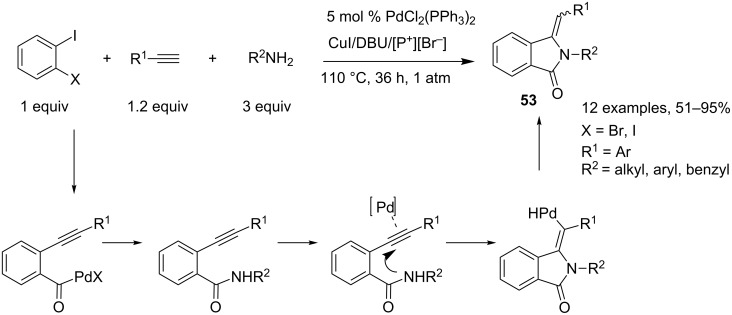
Synthesis of isoindolin-1-one derivatives through a sequential Sonogashira coupling/carbonylation/hydroamination reaction.

Based on this concept, Alper and coworkers reported the synthesis of isoindolin-1-one derivatives **53** through a four-component reaction starting from ortho-dihaloarenes and conducted in phosphonium salt-based ionic liquids (PSILs) with PdCl_2_(PPh_3_)_2_/CuI/DBU as the catalyst system [30]. In this case, the palladium-mediated Sonogashira coupling reaction leading to 1-halo-2-alkynylbenzene derivatives is followed by a carboxyamidation in the presence of carbon monoxide and primary amines [31]. This is followed by an in situ intramolecular hydroamination of the resulting amide on the triple bond, leading to substituted 3-methyleneisoindolin-1-ones in high selectivities in favor of the (*Z*)-isomers (Scheme 24).

**Scheme 25:**
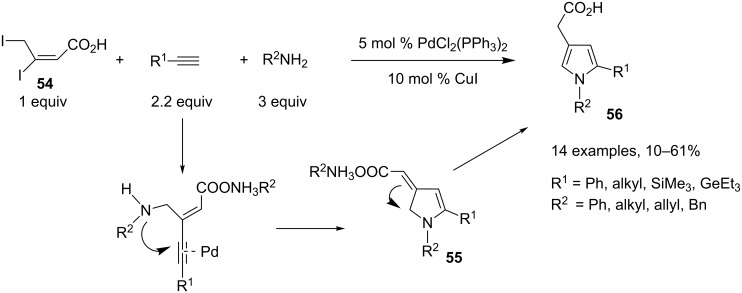
Synthesis of pyrroles through an allylic amination/Sonogashira coupling/hydroamination reaction.

A palladium-mediated three-component process for the preparation of substituted pyrroles involving a dihalogeno substrate and a sequential Sonogashira coupling followed by an hydroamination was developed by Duchêne and Parrain [32]. In this one-pot sequence, the first reaction is an allylic amination between the 3,4-diiodobut-2-enoic acid (**54**) and a primary amine, which can be in competition with the intramolecular lactonization reaction. The best yields of the expected pyrroles were obtained when the three-component reaction was conducted, with five equivalents of the amine partner, at room temperature in DMF, with PdCl_2_(PPh_3_)_2_/CuI as the catalyst system. The initial C–N allylic amination, followed by a Sonogashira cross-coupling and an intramolecular hydroamination furnished a dihydroexoalkylidene pyrrole **55**, which rearranges into pyrrole **56**. This Pd/Cu-mediated three-component approach is influenced by the nature of the nitrogen nucleophile, and the reaction failed with tosylamine and benzylcarbamate, whereas aryl-, alkyl- and benzylamines were used successfully in this reaction (Scheme 25).

A three-component reaction involving in the first step a Sonogashira coupling of *o*-haloanilines **57** with terminal alkynes and leading to *o*-alkynylaniline intermediates **58** was developed by Lu and co-workers [33]. This one-pot reaction is based on a stepwise synthesis of indole derivatives reported by Cachi’s group, and involves, in the last step, a palladium-mediated cyclization of *o*-alkynylaniline derivatives in the presence of aryl halides [34]. In this process, oxidative addition of the aryl halide to the Pd(0) catalyst generates an organopalladium reagent, which activates the alkyne moiety towards nucleophilic attack of the amino group. A reductive elimination generates the indole derivatives **59**.

**Scheme 26:**
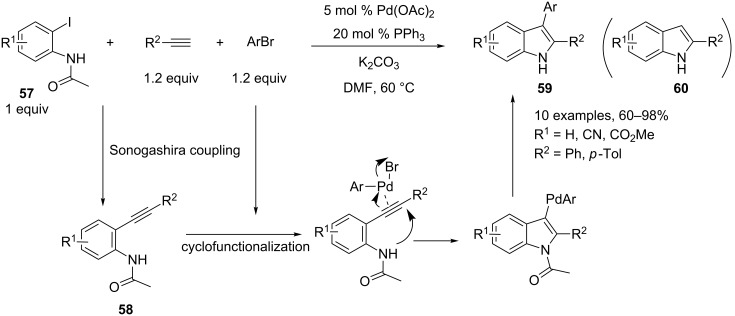
Synthesis of indoles through a Sonogashira coupling/cyclofunctionalization reaction.

In this one-pot three-component reaction, the same palladium complex catalyzes the Sonogashira coupling and the cyclofunctionalization reaction. However, the presence of a strong electron-withdrawing substituent on the amino group is needed for the intramolecular cyclization reaction. Therefore, a protocol for a copper-free Sonogashira coupling was developed in order to suppress the concurrent formation of 2-substituted indoles **60** by direct cyclization of *o*-alkynylaniline intermediates under the classical Sonogashira reaction conditions. Interestingly, aryl bromides were used as a third partner and may be added at the beginning of this one-pot reaction since no competition between the Sonogashira coupling with these substrates and iodoanilides is observed. A variety of 2,3-disubstituted indoles **59** were obtained under mild conditions in good yields (Scheme 26).

**Scheme 27:**
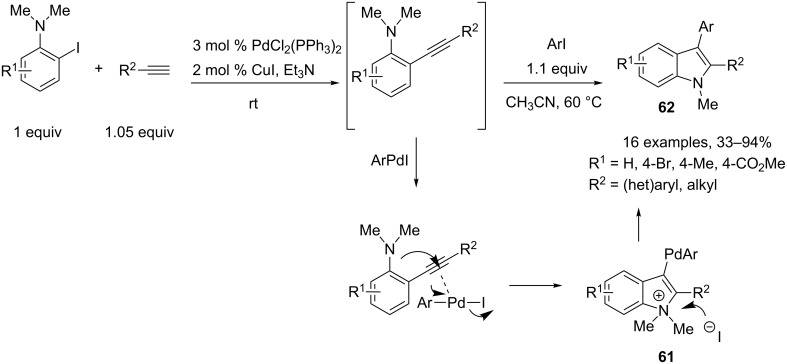
Synthesis of indoles through a one-pot two-step Sonogashira coupling/cyclofunctionalization reaction.

A similar three-component reaction was further developed under microwave irradiation by Larock and coworkers [35]. In this case, *N*,*N*-dimethyl-2-iodoanilines, terminal alkynes and various aryl iodides were involved in the reaction due to the high nucleophilicity of the *N*,*N*-dialkylamino moiety. Here, the reaction needs to be performed in two steps, the aryl iodide in acetonitrile being added after the completion of the first Sonogashira coupling reaction. Regarding the mechanism of the reaction, the intramolecular attack of the amino nucleophile affords here indolium species **61**. Removal of a methyl group by the iodide anion generated in situ, followed by reductive elimination allows the preparation of various 2,3-disubstituted indole derivatives **62** (Scheme 27).

### Amines as coupling partners through Buchwald–Hartwig amination

**Scheme 28:**
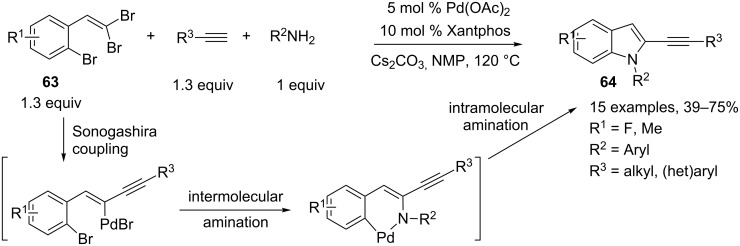
Synthesis of α-alkynylindoles through a Pd-catalyzed Sonogashira/double C–N coupling reaction.

Other strategies used for the palladium-mediated three-component preparation of substituted indole derivatives involve an efficient Buchwald–Hartwig amination as the key step. Xi and co-workers developed an elegant one-pot synthesis of 2-alkynylindoles **64** involving *o*-bromo-(2,2-dibromovinyl)benzenes **63**, arylamines and terminal alkynes as starting partners [36]. It should be noted that the three components are present at the same time in the reaction system and the best results for this Pd-catalyzed tandem Sonogashira/double C–N coupling reaction were obtained when Pd(OAc)_2_ was used as the catalyst along with a bulky bidentate phosphine ligand such as Xantphos in the presence of Cs_2_CO_3_ as base. Most likely, the reaction proceeds through a Pd-catalyzed Sonogashira coupling leading to a mono-alkynylated product, followed by an intermolecular Buchwald–Hartwig amination and a subsequent intramolecular amination. This Pd-catalyzed tandem coupling reaction allows the preparation of a variety of 2-alkynylindoles **64** (Scheme 28).

**Scheme 29:**
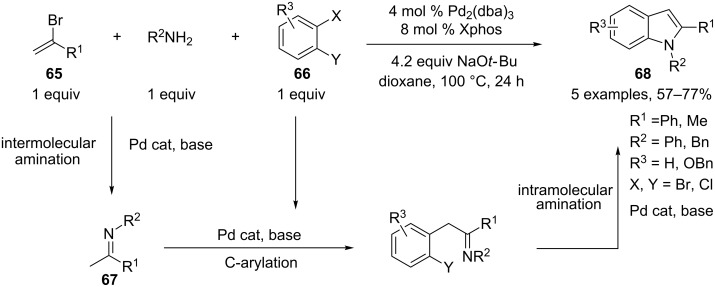
Synthesis of indoles through a Pd-catalyzed sequential alkenyl amination/C-arylation/N-arylation.

An elegant three-component process based on a Pd-catalyzed cascade sequence, involving an alkenyl amination, a C-arylation and a subsequent intramolecular N-arylation, was developed by Barluenga and coworkers for the preparation of indole derivatives **68** [37]. Here, equimolecular amounts of haloalkene **65**, *o-*dihaloarene **66**, and amines are mixed at the start of the reaction. The higher reactivity of the haloalkene toward oxidative addition with palladium, when compared to the haloarene, allowed the unique formation of the imine intermediate **67**. This was followed by the formation of the corresponding aza-allylic anion by deprotonation in basic media. A subsequent Pd-mediated intermolecular alkylation with the dihalogeno substrate followed by an intramolecular N-arylation furnished 2-substituted indoles **68**. In this cascade reaction, the palladium catalyst intervenes in three different coupling reactions: Intermolecular N-alkenylation, C-arylation and intramolecular N-arylation (Scheme 29).

**Scheme 30:**
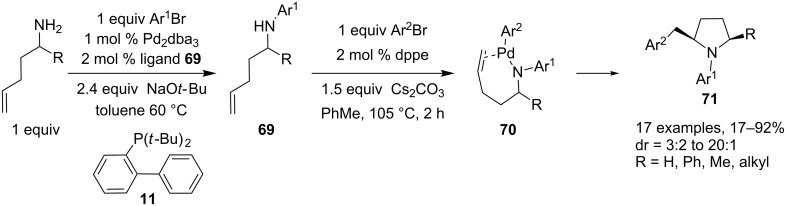
Synthesis of *N*-aryl-2-benzylpyrrolidines through a sequential N-arylation/carboamination reaction.

The palladium-mediated amination reaction coupled with a nitrogen**–**carbon bond-forming reaction was also used for the stereoselective synthesis of *N*-aryl-2-benzylpyrrolidines **71** starting from linear 4-pentenylamine and its derivatives [38]. In this tandem reaction, two different aryl bromides are sequentially added to the primary aliphatic amine in the presence of a palladium(0) catalyst. The first selective, Pd-catalyzed mono-N-arylation leading to the corresponding γ-(*N*-arylamino)alkenes **69** is followed by a carboamination reaction, developed by the same group, after addition of the second aryl bromide [39]. A plausible mechanism for this cyclization/coupling reaction involves formation of intermediate **70** by reaction of the organopalladium complex with the newly formed γ-(*N*-arylamino)alkene **69**. A *syn*-insertion of the alkene into the Pd–N bond in **70** followed by reductive elimination furnishes *N*-aryl-2-benzylpyrrolidine derivatives **71**. In this process, both reactions are catalyzed by zerovalent palladium and the choice of the phosphine ligand for the N-arylation of amines and the carboamination reactions is of great significance and an in situ modification of the catalyst by phosphine ligand exchange was necessary to achieve the selective diarylation in good yields (Scheme 30).

**Scheme 31:**
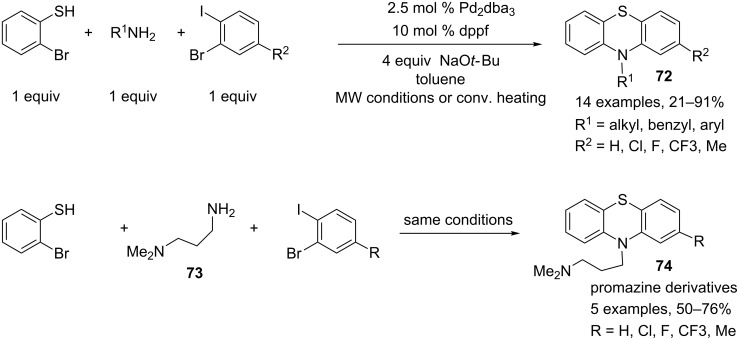
Synthesis of phenothiazine derivatives through a one-pot palladium-catalyzed double C–N arylation in combination with a S-arylation.

A three-component reaction involving a palladium-catalyzed double N-arylation in combination with a S-arylation in a single operation was developed for the preparation of phenothiazine derivatives **72** starting from primary amines, 2-bromothiophenol and substituted 1-bromo-2-iodobenzenes [40]. Ferrocene ligands, such as dppf, and Pd_2_dba_3_ as the palladium source were found to be the most suitable and efficient catalyst systems for the preparation of a series of phenotiazine derivatives. This one-pot procedure worked with a wide variety of primary amines including allyl-, benzyl-, alkyl- and arylamines, and antipsychotic promazine as well as some analogues **74** were synthesized when 3-(dimethylamino)-1-propylamine (**73**) was used as the amine component (Scheme 31).

### Amines as coupling partners through a Pd-mediated allylic amination

**Scheme 32:**
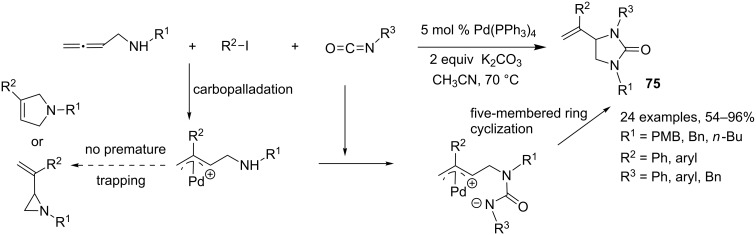
Synthesis of substituted imidazolidinones through a palladium-catalyzed three-component reaction of 2,3-allenyl amines, organic halides and isocyanates.

The allene carbopalladation process with organic halides is known to generate a π-allylpalladium intermediate, which can be trapped by intermolecular carbo- or heteronucleophiles to produce the corresponding three-component adduct. This strategy was used by Ma and coworkers for the selective preparation of five**-**membered nitrogen heterocycles starting from allene-bearing nucleophilic centers [41]. In this context, the same authors developed a new synthesis of substituted imidazolidinones **75** through a palladium-catalyzed, three-component reaction of 2,3-allenylamines, organic halides and isocyanates [42]. In this process, there is first a carbopalladation of the functionalized allene with the aryl iodide, followed by reaction of the internal aza-nucleophile with the highly electrophilic isocyanate derivative, before premature trapping of the initially formed π-allylpalladium intermediate that would lead to 2,5-dihydropyrrole or vinylic azacyclopropane derivatives. This is followed by a five**-**membered ring cyclization leading to polysubstituted imidazolidinones **75** in rather good yields and excellent selectivity (Scheme 32).

**Scheme 33:**
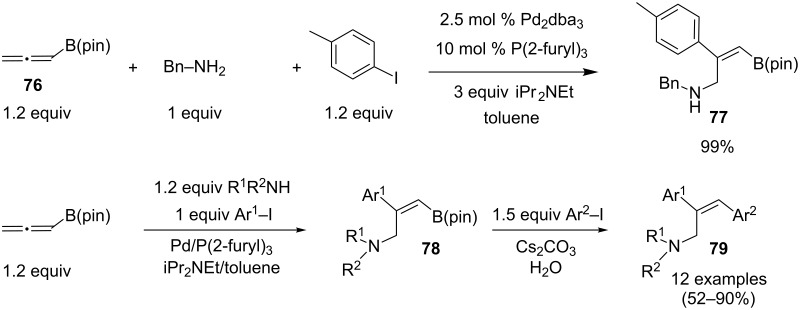
Synthesis of 2,3-diarylated amines through a palladium-catalyzed four-component reaction involving an allenylboronate pinacol ester.

A conceptually related strategy was developed by Yoshida, Itami and Tonogaki [43]. In this case, the palladium-catalyzed allenation with an aryl iodide is performed on the allenylboronate pinacol ester **76** in the presence of benzylamine to afford the functionalized alkenylboronate **77** in quantitative yields and with complete regio- and stereoselectivity. A four-component reaction was further developed through an in situ post C–B arylation by adding a second aryl iodide, with Cs_2_CO_3_ and water, to the newly formed alkenylboronate **78**. The subsequent Suzuki–Miyaura coupling led to the formation of 2,3-diarylated amines **79** and the best results were obtained with secondary amines, the remaining N–H functionality interfering with the C–B arylation step with primary amines as coupling partners (Scheme 33).

**Scheme 34:**
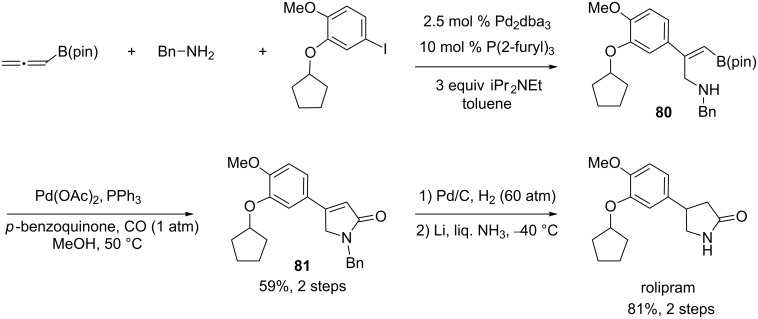
Synthesis of rolipram involving a Pd-catalyzed three-component reaction.

This palladium-catalyzed three-component coupling was applied to the synthesis of rolipram, which is a phosphodiesterase-4 inhibitor. In this process, the Pd-mediated three-component reaction that gives access to the alkenylboronate **80** was followed by a palladium-mediated carbonylative cyclization reaction. Hydrogenation of the resulting unsaturated lactam **81** and removal of the *N-*benzyl group afforded rolipram (Scheme 34).

**Scheme 35:**
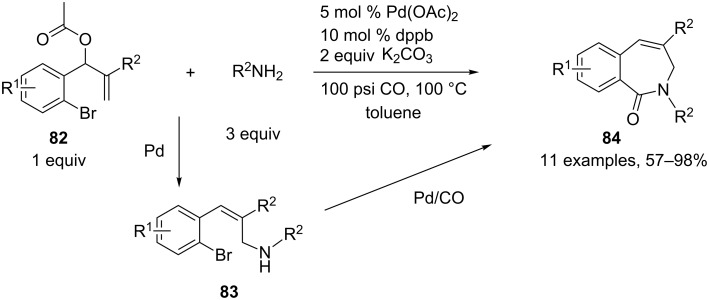
Synthesis of seven-membered ring lactams through a Pd-catalyzed amination/intramolecular cyclocarbonylation.

Alper and coworkers developed several multicomponent approaches for the synthesis of nitrogen-containing heterocycles based on a palladium-mediated carbonylation reaction [44]. An interesting, related strategy for the preparation of unsaturated seven-membered ring lactams **84**, starting from a Baylis–Hillman adduct bearing an aryl bromide moiety **82**, with primary amines and carbon monoxide, was developed by the same group [45]. The sequence involves first a selective palladium(0)-catalyzed amination on the Baylis–Hillman acetates with primary amines leading to allylic amines **83**. This is followed by oxidative addition of the palladium species to the aryl bromide, which undergoes CO insertion to form the corresponding acylpalladium, which in turn is intercepted by the allylamine to give, after reductive elimination, the seven-membered ring lactams **84** in good to excellent yields. A wide range of amine components are compatible with this one-pot procedure (Scheme 35).

## Conclusion

In summary, this review highlights the usefulness of amines as key building blocks in the development of Pd-mediated multicomponent approaches to polyfunctionalized nitrogen acyclic or cyclic compounds. Amines may be involved in several bond-forming transformations, including aza-Michael additions, hydroaminations of alkynes, Buchwald–Hartwig aminations, and allylic aminations, thereby allowing the creation of several covalent bonds in a single operation. Imine derivatives are also of high synthetic value as they may act either as electrophilic or nucleophilic partners. It is expected that further useful, new multicomponent processes in which amines play a central role will be developed in the near future.
